# Nasopharyngeal Ventilation Compared to Facemask Ventilation: A Prospective, Randomized, Crossover Trial in Two Different Elective Cohorts

**DOI:** 10.7759/cureus.39049

**Published:** 2023-05-15

**Authors:** Rainer Lenhardt, Ozan Akca, Detlef Obal, Jerrad Businger, Elisabeth Cooke

**Affiliations:** 1 Anesthesiology, University of Louisville, Louisville, USA; 2 Anesthesiology, Johns Hopkins University, Baltimore, USA; 3 Anesthesiology, Stanford University, Stanford, USA

**Keywords:** endotracheal tube, placement of endotracheal tube (ett), nasopharyngeal ventilation, preoxygenation, mask ventilation, supraglottic airway

## Abstract

Background: Facemask ventilation is routinely used to preoxygenate patients before endotracheal intubation during anesthesia induction or to secure ventilation in patients with respiratory insufficiency. Occasionally, facemask ventilation cannot be performed adequately. The placement of a regular endotracheal tube through the nose into the hypopharynx may be a valid alternative to improve ventilation and oxygenation before endotracheal intubation (nasopharyngeal ventilation). We tested the hypothesis that nasopharyngeal ventilation is superior in its efficacy compared to traditional facemask ventilation.

Methods: In this prospective, randomized, crossover trial, we enrolled surgical patients requiring either nasal intubation (cohort #1, n = 20) or patients who met “difficult to mask ventilate” criteria (cohort #2, n = 20). Patients in each cohort were randomly assigned to receive pressure-controlled facemask ventilation followed by nasopharyngeal ventilation or vice versa. The ventilation settings were kept constant. The primary outcome was tidal volume. The secondary outcome was the difficulty of ventilation, measured using the Warters grading scale.

Results: Tidal volume was significantly increased by nasopharyngeal ventilation in cohort #1 (597 ± 156 ml vs.462 ± 220 ml, p = 0.019) and cohort #2 (525 ± 157 ml vs.259 ± 151 ml, p < 0.01). Warters grading scale for mask ventilation was 0.6 ± 1.4 in cohort #1, and 2.6 ± 1.5 in cohort #2.

Conclusion: Patients at risk for difficult facemask ventilation may benefit from nasopharyngeal ventilation to maintain adequate ventilation and oxygenation before endotracheal intubation. This ventilation mode may offer another option for ventilation at induction of anesthesia and during the management of respiratory insufficiency, especially in the setting of “unexpected” ventilation difficulty.

## Introduction

Preoxygenation with facemask ventilation is a routine process during anesthesia induction before tracheal intubation. In addition, facemask ventilation is essential in the management of acute respiratory insufficiency until the patient’s airway is secured using an endotracheal tube (ETT). Occasionally, facemask ventilation cannot be performed adequately, and intubation may fail (“cannot ventilate, cannot intubate” situation). Failed airway management may result in increased morbidity and mortality [1-4].

Difficult facemask ventilation has been reported to occur 8.9% of the time in a retrospective analysis of 1399 surgical patients and 14% in 557 obese patients [5]. Difficult facemask ventilation is defined as failure to maintain adequate tidal volumes and subsequent desaturation ensues [1]. Reasons for failure of facemask ventilation are an inadequate seal of the mask over the face, redundant oropharyngeal tissue causing increased airway resistance, or a combination of both [5]. Several factors have been identified to predict difficult facemask ventilation, including facial hair, large neck circumference, morbid obesity, edentulism, obstructive sleep apnea, higher Mallampati class, older age, history of difficult intubation, and certain maxilla-mandibular pathologies [6-9].

The American Society of Anesthesiologists (ASA) difficult airway algorithm suggests using a supraglottic airway as an alternative in this situation [10]. However, a specifically designed supraglottic airway device may not always provide successful airway insertion and ventilation [11].

In addition to the ASA algorithm, nasopharyngeal ventilation with an ETT has been suggested as a rescue ventilation approach while awaiting alternative difficult airway equipment [12]. Ventilation through an extra/supraglottic tracheal tube was also described for airway control after deep extubation [13].

The tested technique was accomplished by inserting an ETT nasally to the level of the hypopharynx, while simultaneously sealing the mouth and nares to achieve successful positive pressure ventilation. The described technique can also be utilized for patients predicted to have difficult mask ventilation. However, the efficacy and safety of this method have not been systematically evaluated. The primary objective of the study was the efficacy of a nasopharyngeal airway compared to facemask ventilation. Thus, we set out to test the hypothesis that ventilation via a nasally placed ETT provides larger expiratory tidal volumes (eTVs) than routine facemask ventilation.

## Materials and methods

Study design

**Figure 1:**
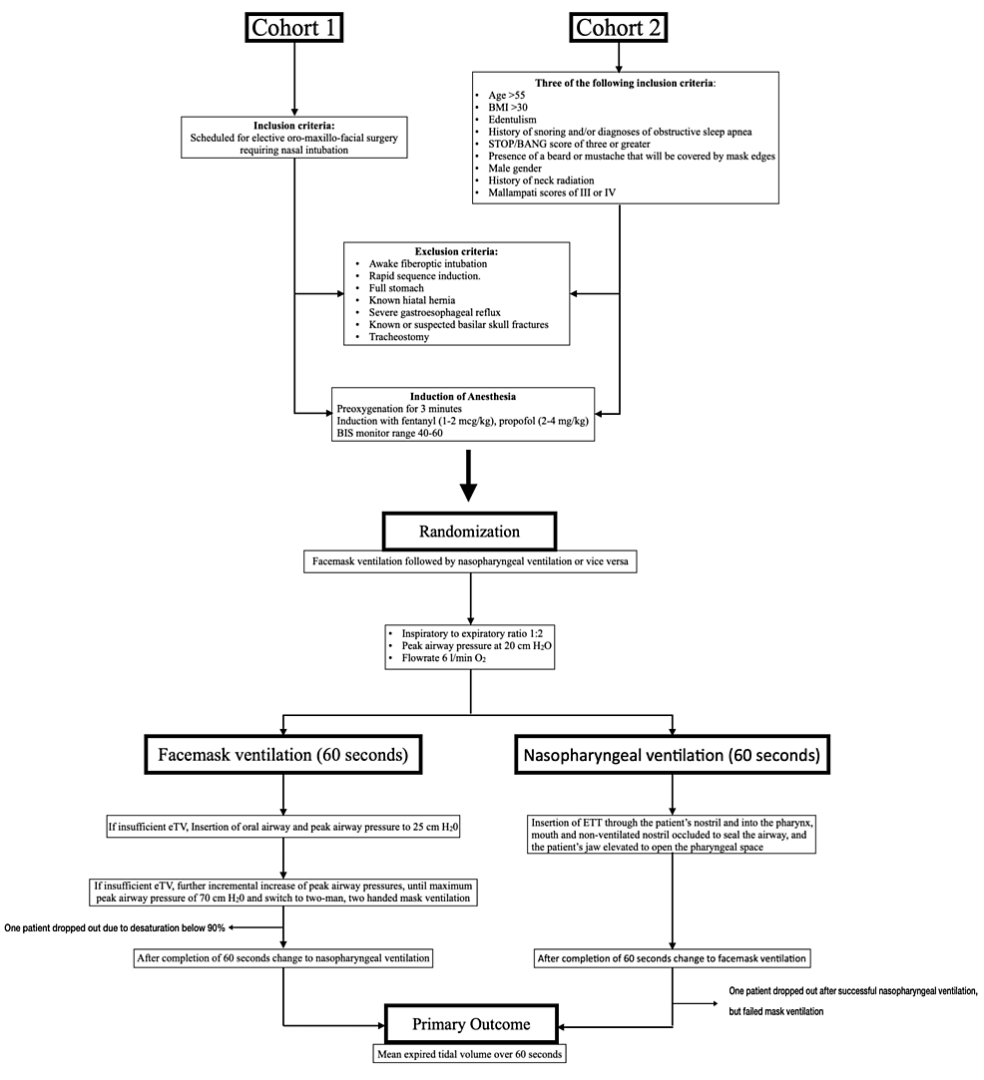
Flow chart of patients and study design eTV: end-tidal volume; ETT endotracheal tube; BIS: bispectral index.

The study was designed as a crossover, randomized controlled trial comparing facemask ventilation (MV, control) with nasopharyngeal ventilation (NPV, treatment) for preoxygenation in two different cohorts undergoing general anesthesia (Figure 1). The first cohort consisted of patients who required nasal intubation prior to surgery. The second cohort consisted of patients with anticipated difficult facemask ventilation according to predefined criteria (see below). The study was registered at ClinicalTrials.gov (NCT02018146) and conducted under the Good Clinical Practice guidelines.

This study was approved by the Human Studies Committee of the University of Louisville and the University of Louisville Hospital Research Integrity Office (IRB # 13.0570). All subjects were informed preoperatively and signed written informed consent. The study was performed at the University of Louisville Hospital between June 2017 and May 2020.

Participants

Patients aged 18-81 years with ASA physical status I to III were included in the study in two independent cohorts. In the first cohort, 20 patients scheduled for elective oromaxillofacial surgery requiring nasal intubation were enrolled (cohort #1). The second cohort (cohort #2) included 20 patients scheduled for elective surgery with predicted difficult facemask ventilation. Criteria for difficult facemask ventilation were as follows: age > 55 years, BMI > 30, edentulism, history of snoring and/or diagnoses of obstructive sleep apnea, STOP-Bang score of three or greater, presence of a full beard that will be covered by mask edges, male gender, history of neck radiation, and Mallampati scores of III or IV [5,6]. Patients who met three of the listed criteria were considered to have potentially difficult facemask ventilation.

Patients were excluded from this study if awake fiberoptic intubation or rapid sequence induction was required. In addition, patients with a full stomach, known hiatal hernia, severe gastroesophageal reflux, tracheostomy, and known or suspected basilar skull fractures were excluded.

Intervention

The oropharyngeal view was graded according to the modified Mallampati classification [14]. The patient’s nares were prepped prior to induction with two sprays of nasal decongestant bilaterally (NeoSynephrine Nasal 0.5%, Asher, Lenexa, KS). Standard monitoring was applied before induction, including ECG, pulse oximetry, noninvasive blood pressure monitoring, and end-tidal gas monitoring, In addition, the bispectral index (BIS) was monitored. The patient’s head was rested on a disposable anesthesia pillow. The patient was preoxygenated until an end-tidal oxygen concentration of 90% or higher was achieved. General anesthesia was induced using fentanyl (1-2 mcg/kg) and propofol (2-3 mg/kg). Once the BIS reached a range of 40 to 60, the study was started. Additional propofol was administered via propofol infusion or bolus dose(s) to maintain the same depth of anesthesia throughout the protocol.

During the entire study period, we measured and recorded end-tidal oxygen and carbon dioxide concentrations for each breath as well as the current BIS. Measured eTVs were recorded for each breath during a 60-second period, and ventilation was considered sufficient when eTVs were similar or greater than 2 ml/kg (ideal body weight). In both study groups, ventilation was maintained with a peak inspiratory pressure (PIP) of 20 cm H20 set on the adjustable pressure-limiting (APL) valve and a respiratory rate of 10 breaths per minute with an I:E ratio of approximately 1:2 at a gas flow rate of 6 L/min. The ventilation conditions were strictly enforced by a study coordinator using a stopwatch.

Facemask ventilation: When randomized to facemask ventilation, ventilation was initiated with a medium adult facemask (Smiths Medical International Ltd., Hythe, Kent, UK), one-handed, with 100% oxygen. Ventilation was confirmed by the presence of an end-tidal CO2 waveform, chest rise and fall, eTV, and oxygen saturation on the pulse oximeter. If facemask ventilation resulted in insufficient eTV (<2 ml/kg), an oral airway was initially inserted, and the PIP subsequently increased to 25 cm H20. The PIP would increase as needed until sufficient eTV (≥2ml/kg) was achieved, or a maximum of 70 cm H2O was reached. If sufficient facemask ventilation was not achieved despite increasing PIP, the investigator would switch to two-handed facemask ventilation. Once 60 seconds of one ventilation mode was completed, the patient was switched to the second ventilation method according to randomization. A drop in arterial oxygen saturation below 90% would initiate nasopharyngeal ventilation as a rescue measure.

Nasopharyngeal ventilation: When randomized to nasopharyngeal ventilation, a lubricated and properly sized regular endotracheal tube (ETT# 6 in women and ETT #7 in men; Parker Flex-Tip, Parker Medical, Highlands Ranch, CO) was inserted through the patient’s nostril and into the pharynx. The depth of ETT insertion was determined prior to induction by measuring the distance from the patient’s nostril to the angle of the jaw, which has been shown to approximate the nasopharyngeal distance [15]. The pilot balloon remained deflated throughout nasopharyngeal ventilation.

The anesthesia circuit was attached to the ETT connector, the mouth and non-ventilated nostrils were occluded to seal the airway, and the patient’s jaw was elevated to open the pharyngeal space. The lungs were ventilated using the same criteria as those mentioned for facemask ventilation. The ventilation mode was maintained for 60 seconds, and the measured eTV was recorded for each breath.

Once the randomly assigned second ventilation mode was completed, the patient was paralyzed with rocuronium (0.6 mg/kg). Nasopharyngeal ventilation or facemask ventilation (i.e., second randomly assigned ventilation mode) was continued for three minutes. At this time, the patient’s trachea was intubated using direct laryngoscopy. A final inspection of the larynx and evaluation of any bleeding were performed. Subsequently, the study period was concluded. Four attending anesthesiologists performed all mask and nasopharyngeal ventilations, with an average of 20 or more years of experience in difficult mask management, and one anesthesiologist had approximately seven years of experience.

Outcome Measurements

The primary outcome measure was eTV in mL during facemask and nasopharyngeal ventilation.

Secondary outcomes were the difficulty of facemask ventilation, switch of mask ventilation to nasopharyngeal ventilation, nose bleeding, glottis view, the distance of the tip of the ETT to vocal cords, and ETT mark at nasal opening.

Preoperative Assessment

Patient age, sex, weight, height, body mass index (BMI), and ASA physical status were recorded. The patient’s airway was evaluated, and the following variables were recorded: dentition, history of obstructive sleep apnea (OSA), presence of full beard, Mallampati score, history of head/neck radiation, STOP-Bang score, and the distance between the nostril and the angle of the jaw.

Post-induction Assessment

All the measured parameters were recorded by an investigator who was not included in the study. eTV was recorded breath-by-breath from the anesthesia machine (Apollo, Dräger US, Telford, PA) for 60 seconds during both modes of ventilation. Insertion of an oral airway, increase in PIP, or two-hand facemask ventilation were recorded. The difficulty of facemask ventilation was determined by the Warters grading scale using four grades [16]. At grade one of the scale, patients could be ventilated by mask only, grade two required an oral airway or other adjuvants, grade three meant difficult to ventilate, and grade four stood for unable to ventilate. Additionally, the occurrence of nasal bleeding was assessed at the end of the study. At the end of nasopharyngeal ventilation, the distance between the tip of the nasopharyngeal tube and the glottis was measured using a disposable tracheoscope (aScope 3, Ambu Inc., Columbia, MD). The tracheoscope was advanced through the nasopharyngeal tube to the glottis and then withdrawn to the tip of the tube. The difference in distance between the glottis and the tip of the tube was measured in centimeters. Subsequently, the tracheoscope was removed. Direct laryngoscopy was performed to advance the nasopharyngeal tube into the trachea. During intubation, the patient’s pharynx was assessed for bleeding. Bleeding was defined as none (no presence of blood), mild (small spots of blood in the hypopharynx), moderate (bloody secretions in the hypopharynx requiring suctioning), and severe (appreciable ongoing bleeding in the hypopharynx). The laryngeal view was obtained and graded according to the method described by Cormack and Lehane [17].

Sample Size Calculation

Preliminary data have shown that nasopharyngeal ventilation may improve the applied eTV compared to facemask ventilation.

Sample size analysis: A within-group difference of 50 ml eTV was considered to be clinically significant in our study because such a difference would be equivalent to a 10% increase in eTV during nasopharyngeal ventilation. To detect an increase of 10% in eTV, 16 patients were required with 90% power at a significance level of 0.05; thus, 20 patients in each cohort were enrolled for this crossover trial. eTV and airway pressures were compared using paired t-tests. Data are presented as mean ± standard deviation.

Randomization and Sequence Generation

The patients were randomly assigned to receive either facemask ventilation followed by nasopharyngeal ventilation, or vice versa. Randomization was based on computer-generated codes maintained in sequentially numbered opaque envelopes.

## Results

Morphometric data and predictive indices for difficult facemask ventilation were assessed (Table 1). There were more male than female patients in each cohort. BMI and weight were higher in cohort #2 (i.e., potentially difficult facemask ventilation). According to this definition, all patients in cohort #2 had three or more risk factors for difficult facemask ventilation. Specifically, four patients had three risk factors, one patient had four, six patients had five, four patients had six, three patients had seven, and two patients had eight risk factors. Two patients in cohort #1 could not be ventilated by a facemask. In one patient, arterial oxygen saturation decreased below 90% despite oral airway, increased inspiratory pressure, and two-hand ventilation. The patient was successfully intubated after succinylcholine administration. Nasopharyngeal ventilation was not provided. A second patient could not be ventilated with a facemask after successful nasopharyngeal ventilation in the first half of the study. Therefore, a nasopharyngeal ETT was reinserted into the nasopharynx to re-institute adequate eTV. These two patients were not included in the analysis because the main study outcomes could not be collected. Thus, 18 patients completed the study in cohort #1.

**Table 1:** Morphometric data (mean ± SD) Data presented as the number of subjects unless otherwise noted. Cohort #1: Elective oromaxillofacial surgery requiring nasal intubation. Cohort #2: Patients with predicted difficult mask ventilation under a predefined set of criteria scheduled for elective surgery. ASA: American Society of Anesthesiologists.

	"Requiring nasal intubation" (Cohort #1, n = 20), mean ± SD or frequency	"Difficult to mask ventilate” criteria (Cohort #2, n = 20), mean ± SD or frequency
Age (years)	51 ± 23	53 ± 12
Weight (kg)	88 ± 27	100 ± 30
Height (cm)	172 ± 15	174 ± 12
Body mass index (BMI > 30)	6/20	10/20
Male/female	16/20	14/20
ASA class I/class II/class III	1/10/9	0/6/14
No teeth missing/some teeth missing/edentulous	10/5/5	9/8/0
STOP-Bang score ≥ 3	14/20	16/20
Presence of a full beard	6/20	10/20
History of neck radiation	1/20	0/20
Modified Mallampati score 3 &4	12/20	12/20

The eTVs were significantly increased during nasopharyngeal ventilation in cohort #1 (597 ± 156 ml vs. 462 ± 220 ml, p = 0.019) and cohort #2 (525 ± 157 ml vs. 259 ± 151 ml, p < 0.001). The absolute difference in cohort #1 was 135 ml or a 35% increase in eTV. In cohort #2, the absolute difference was 266 ml, reflecting a 103% increase in eTV (Table 2). The median (interquartile range) of the Warters grading scale for facemask ventilation was 0 (0, 1.5) in cohort #1, and 2.9 (0.8, 4.3) in cohort #2. Apart from two patients in cohort #1, no significant desaturation was observed. No significant nose bleeding was detected in either cohort during the study period. Six patients in cohort #1 and two patients in cohort #2 experienced minor bleeding, which was relieved by nasal vasoconstricting decongestant spraying. Two patients in cohort #1 and one patient in cohort #2 had moderate bleeding. All the patients were successfully intubated at the end of the study period.

**Table 2:** Primary outcome (ventilation) of the two cohorts ^a^ Student’s t-test; eTV: end-tidal volume.

	Nasopharyngeal ventilation (eTV in ml) (mean ± SD)	Mask ventilation (eTV in ml) (mean ± SD)	Difference between the mask and nasopharyngeal ventilation (eTV in ml) (mean, CI)	P-value
"Requiring nasal intubation" - Cohort #1 (n = 18)	597 ± 156	462 ± 220	135 (26,244)	^a^0.019
"Difficult to mask ventilate” criteria - Cohort #2 (n = 20)	525 ± 157	259 ± 151	266 (187,329)	^a^<0.001

The distance of the ETT tip from the vocal cords was 4 ± 2 cm in cohort #1 and 4 ± 3 cm in cohort #2. The glottis view per classic Cormack and Lehane classification scored 2 on average in both cohorts (Table 3).

**Table 3:** Qualitative & quantitative secondary outcomes ETT: endotracheal tube.

Outcomes	"Requiring nasal intubation", Cohort #1 (n = 18) (median (IQR), mean ± SD or frequency)	"Difficult to mask ventilate” criteria, Cohort #2 (n = 20) (median (IQR), mean ± SD or frequency)
Warters grading scale (mask ventilation)	0 (0, 1.5)	2.9 (0.8, 4.3)
Mask ventilation-nasopharyngeal ventilation switch	1 of 20	0 of 20
Nose bleeding (none/mild/moderate/severe)	12/6/2/0	17/2/1/0
Glottis view (Cormack/Lehane)	2 ± 1	2 ± 1
Distance ETT to vocal cords during nasopharyngeal mask ventilation (cm)	4 ± 2	4 ± 3
Distance nostrils to jaw (cm)	12 ± 1	12 ± 3
ETT mark at nasal opening (cm)	Not measured	15 ± 2

## Discussion

In this study, we aimed to demonstrate the role of nasopharyngeal ventilation in providing larger tidal volumes compared to facemask ventilation in anesthetized, but not paralyzed, elective surgery patients with and without potentially difficult facemask ventilation. Specifically, we compared nasopharyngeal ventilation with facemask ventilation in two different cohorts of patients: cohort #1, patients requiring nasal intubation for their surgeries; and cohort #2, patients with potentially difficult facemask ventilation. We proved our hypothesis that nasopharyngeal ventilation resulted in clinically and statistically higher tidal volumes of ventilation in both cohorts.

Most patients can be ventilated using facemasks, without additional airway equipment. However, there is a subset of patients who will turn out to be difficult to ventilate with a facemask [7]. In this patient population, additional measures may mitigate the difficulty of facemask ventilation, such as oral airways, nasal tubes, or applying higher PIP. Despite using auxiliary measures, some patients remain to be very difficult to ventilate with a facemask due to an inadequate seal, resulting in a major air leak or excessive airway resistance [10].

Using the nasal route for ventilation in anesthetized patients has been shown to be more effective when compared with the oral route, i.e., using special oral and nasal masks [18]. Likewise, Jiang et al. demonstrated a higher effectiveness of breathing through nasal routes compared to oral routes in anesthetized patients [19]. These results suggest that the nasal route may be more suitable for ventilation in anesthetized patients than oral breathing. This is further supported by a case report that showed the superiority of a nasopharyngeal tube over facemask ventilation in a difficult-to-mask patient [20]. To our knowledge, this study is the first to systematically investigate the difference in eTV using a nasopharyngeal airway versus standard care facemask ventilation.

Nasopharyngeal ventilation may be superior to facemask ventilation, as it can potentially overcome and simply bypass upper airway obstruction caused by the soft palate, base of the tongue, and redundant retropharyngeal fatty tissue. Furthermore, it makes it easy to deal with the oral route circumventing facial hair and an edentulous mouth, both of which are known to cause air leaks during facemask ventilation. When considering nasal ventilation through the nasal airway, one can use a soft nasal airway with an adaptor from a regular ETT. However, nasal airways can be too short to face the glottis [20], and they typically do not allow a fiberoptic scope to pass through or cannot be used as blind intubators, such as ETTs. In this study, we used a regular #6 ETT in females and a #7 ETT in males; the ETT was placed in one nostril and advanced to the retropharynx, where it reached proximity to the vocal cords. This technique is typically used for patients undergoing nasal intubation.

Intranasal placement of an ETT may cause epistaxis or nasopharyngeal bleeding; hence, nasal preparation with a vasoconstricting decongestant spray is highly recommended to minimize the risk of bleeding.

We used the nasopharyngeal ventilation technique in two cohorts undergoing elective procedures. We cannot claim this technique under emergency conditions, but it may provide adequate ventilation in emergency situations in patients who are in immediate need of ventilatory support [21] when other supraglottic airways fail to provide this support.

In the first cohort, we enrolled patients who required nasotracheal intubation but did not meet the criteria for difficult facemask ventilation. However, even in patients without a single predictive factor of difficult facemask ventilation, we found an advantage of nasopharyngeal ventilation over facemask ventilation in terms of eTV. In the second cohort, we identified patients who met at least three criteria for difficult facemask ventilation and found an even larger difference in eTV between nasopharyngeal and facemask ventilation.

Our study has several limitations. First, in both patient cohorts, we kept breathing pressures at 20 cm H2O and escalated to the use of oral airways and two-hand facemask ventilation strictly per protocol. Differences between nasopharyngeal and facemask ventilation were different if we had used higher airway pressures and/or oral airways and two-hand facemask ventilation more frequently. In addition, we did not paralyze patients during the two-minute study period. Paralysis may facilitate facemask ventilation and render auxiliary measures unnecessary [22].

Second, not paralyzing may have kept patients spontaneously breathing or regaining spontaneous ventilation in the two-minute study period. However, by using the anesthesia regimen as described in the methods, we did not encounter any spontaneous breathing efforts in the short study period.

Third, blinding of participating anesthesiologists was not possible. Despite the best efforts, provider bias cannot be ruled out. However, the I:E ratio and breathing pressures were kept strictly per protocol, and airway sealing was performed to the best ability of the providers in both group assignments. We did not use preset pressure control ventilator settings. Instead, we manually ventilated the patients in a strict six-second cycle with the APL valve at 20 cm/H20 at a gas flow rate of 6 L/min. This may have enhanced provider bias. However, pre-study training of the investigators showed that there were only minimal differences between manual ventilation and pressure control settings during facemask ventilation.

Fourth, only four attendings participated in the study. Therefore, it may be difficult to generalize our results. The range of expertise of the participating attendants was seven to 32 years. Thus, a wide variety of cumulative anesthesia experiences was used in this study.

Lastly, the insertion of a nasopharyngeal tube may cause nasal bleeding. Despite a significant number of nasal bleedings, the great majority of these bleedings were mild and clinically insignificant, when nasal decongestants and careful nasal insertion were used (Table 3).

## Conclusions

We evaluated a nasopharyngeal airway for ventilation in two cohorts of patients who underwent elective intubation. In both cohorts, we showed higher tidal volumes when using nasopharyngeal ventilation through an endotracheal tube than when using facemask ventilation under a strict ventilation protocol. This ventilation mode may offer another option for ventilation at induction of anesthesia and during the management of respiratory insufficiency, especially in the setting of “unexpected” ventilation difficulty. When using a nasopharyngeal path for ventilation, it may be advantageous to prepare nostrils with vasoconstricting decongestants to avoid excessive nose bleeding.
